# Rising C-Reactive Protein and Procalcitonin Levels Precede Early Complications After Esophagectomy

**DOI:** 10.1007/s11605-015-2745-z

**Published:** 2015-02-07

**Authors:** Sandra H. Hoeboer, A. B. Johan Groeneveld, Noel Engels, Michel van Genderen, Bas P. L. Wijnhoven, Jasper van Bommel

**Affiliations:** 1 Department of Intensive Care, Erasmus Medical Center, Gravendijkwal 230, 3015 CE Rotterdam, The Netherlands; 2 Department of Surgery, Erasmus Medical Center, Rotterdam, The Netherlands

**Keywords:** Esophagectomy, Esophageal cancer, Post-operative complications, Anastomotic leak, Biomarkers, Procalcitonin, C-reactive protein

## Abstract

**Background:**

Elective esophagectomy with gastric tube reconstruction carries a high risk for complications. Early and accurate diagnosis could improve patient management. Increased C-reactive protein (CRP) levels may be associated with any, surgical or infectious, complication and procalcitonin (PCT) specifically with infectious complications.

**Methods:**

We measured CRP and PCT on post-operative days 0, 1, 2, and 3 in 45 consecutive patients. Complications were recorded up to 10 days post-esophagectomy.

**Results:**

Twenty-eight patients developed a post-operative complication (5 surgical, 14 infectious, 9 combined surgical/infectious, including anastomotic leakage), presenting on day 3 or later. Elevated days 2 and 3 and a rise in CRP preceded the diagnosis of general or combined surgical/infectious complications (minimum area under the receiver operating characteristics curve (AUROC) 0.75, *P* = 0.006). Elevated day 3 PCT preceded combined complications (AUROC 0.86, *P* < 0.001). High day 1 and 3 PCT levels preceded anastomotic leakage (minimum AUROC 0.76, *P* = 0.005), as did the day 3 CRP levels and their increases (minimum AUROC 0.78, *P* = 0.002).

**Conclusions:**

This small study suggests that high or increasing CRP levels may precede the clinical diagnosis of general or surgical/infectious complications after esophagectomy. Elevated PCT levels may more specifically and timely precede combined surgical/infectious complications mainly associated with anastomotic leakage.

## Introduction

Early complications after elective esophagectomy and gastric tube reconstruction are associated with increased morbidity and mortality.^1^–^5^ Recognition of patients at risk for complications before presentation of full-blown symptoms could lead to early diagnosis and treatment which may improve outcome. However, the early recognition of complications by clinical characteristics and parameters in individual patients remains difficult, except perhaps for pulmonary complications.^2^,^3^,^6^ Esophagectomy in itself induces a strong inflammatory response, and the value of systemic inflammatory response syndrome (SIRS) criteria fever, leukocytosis, tachypnea, and tachycardia for the early diagnosis of complications is limited.^6^–^8^ On the other hand, inflammatory biomarkers like C-reactive protein (CRP) and procalcitonin (PCT) might be useful in the early diagnosis of not yet clinically symptomatic post-operative complications. Previous studies reported an association between elevated CRP levels and (infectious) complications, sepsis, and mortality after esophagectomy.^8^–^12^ However, CRP levels did not discriminate between surgical and infectious complications, requiring different therapeutic management strategies.^6^,^8^–^10^,^12^,^13^ PCT is an allegedly more specific marker of severe infection and complications after surgery than CRP,^14^–^16^ but the literature is inconclusive in this respect.^17^,^18^ So far, only five studies reported on PCT levels post-esophagectomy,^1^,^11^,^13^,^19^,^20^ of which only two focused on post-operative infectious complications.^11^,^13^ The latter studies suggested that PCT is useful for the diagnosis of infectious complications and discriminating sepsis from SIRS post-esophagectomy. The discriminating ability of PCT for complication subtypes is unknown, however.

We hypothesized that CRP is a sensitive but non-specific marker of developing complications after esophagectomy, while PCT is a more specific marker of developing severe post-operative infections. We thus compared the use of CRP and PCT for early diagnosis of surgical and infectious complications.

## Patients and Methods

This prospective observational study, approved by the Medical Ethical Committee of the Erasmus Medical Centre (MEC-2010-199), was conducted between September 2011 and December 2012. Forty-five consecutive adult patients were included after giving written informed consent prior to surgery. We did not perform a power analysis for this proof of principle study. Because of competing studies and activities, this proof of principle study was limited in time, and therefore, we could include only 45 patients in the time interval indicated. Esophagectomy and gastric tube reconstruction was performed by the transthoracic or transhiatal approach.^21^ The gastric tube reconstruction was performed by handsewn end-to-end or semimechanical end-to-side anastomosis.^22^ After admission to the intensive care unit (ICU), patients were taken care of by board-certified intensivists unaware of biomarker results.

### Study Protocol

Upon ICU admission (day 0), baseline patient characteristics were recorded. Disease severity was estimated using the Acute Physiology and Chronic Health Evaluation II (APACHE II) score, and organ failure was calculated by the Sequential Organ Failure Assessment (SOFA) score. The preoperative risk assessment was done by using the American Society of Anesthesiologists (ASA) classification and Portsmouth predictor modification of the Physiological and Operative Severity Score for the enUmeration of Mortality and morbidity (P-POSSUM). Clinical parameters and blood samples for routine laboratory parameters, leukocyte counts, and CRP and PCT levels were collected directly post-operatively on ICU admission (day 0) and in the morning of post-operative days 1, 2, and 3. Leukocyte counts were measured using the Sysmex SE-9000 analyzer (Toa Medical Instruments, Kobe, Japan), and normal values are 3.5–10 × 10^9^/L. CRP was measured by an immunoturbidimetric assay (Modular analytics <P> Roche diagnostics, Mannheim, Germany), and normal values are < 9 mg/L. PCT was measured using the PCT sensitive for the Kryptor compact system (Brahms Diagnostica, Hennigsdorf, Germany). Assays were performed according to the manufacturer’s guidelines, the lower detection limit being 0.02 ng/mL, with an upper limit in healthy volunteers of 0.05 ng/mL. The functional assay sensitivity (FAS) of this test is 0.06 ng/mL, with an intra-assay coefficient of variation (CV) and inter-assay CV of < 6 % in samples containing > 0.3 ng/mL.

### Definitions

All complications up to 10 days post-esophagectomy as decided by attending physicians were recorded, only if additional medical or surgical treatment was required, notably grade 2 or higher on the Accordion Severity Grading System.^4^,^5^ The definitions of complications used in this study are depicted in Table 1. The 10-day cutoff was chosen based on a previous study from this group.^12^ Infections were defined according to the International Sepsis Forum Consensus Conference criteria,^23^ as agreed upon by the attending intensivists. Diagnostic imaging and collection of specimens and blood for microbial culture were left at the attending intensivist’s discretion. Specimens were processed according to standardized culture protocols, and Gram stains were prepared. Cultures reflecting colonization rather than infection were excluded from final analysis. For example, blood cultures containing coagulase-negative staphylococci were considered contaminated if only one bottle showed growth. Because reporting of definite culture results can take several days, the day of specimen collection was considered the day of infection diagnosis. Patients were considered to have sepsis when presenting at least two SIRS criteria: body temperature < 36 °C or > 38.3 °C, heart rate > 90 bpm, respiratory rate > 20 breaths/min or mechanical ventilation, and a leukocyte count of either < 4.0 × 10^9^/L or > 12.0 × 10^9^/L, in the presence of a probable or proven infection, according to the American College of Chest Physicians/Society of Critical Care Medicine guidelines.^24^ Shock was defined by a systolic pressure < 90 mmHg or a mean arterial pressure < 60 mmHg for at least 1 h, despite adequate fluid resuscitation, or requirement of vasopressor support to maintain mean arterial pressure. Shock in the presence of sepsis was considered septic shock. We report 30-day mortality.

**Table 1:** Definition of complications

Complication	Definition
Surgical	
Anastomotic leakage	Esophagoenteric leak confirmed by endoscopy or esophageal contrast videography that requires local treatment, surgical treatment, or removal of conduit.
Pleural effusion	Pleural effusion confirmed by radiology that requires drainage.
Chyle leak	Chylomicrons in pleura aspirate or milky discharge from chest tube at initiation of enteral feeding.
Laryngeal nerve palsy	Clinically suspected vocal cord paralysis confirmed by laryngoscopy.
Conduit ischemia/necrosis	Circular conduit ischemia/necrosis confirmed by endoscopy and/or surgically that requires local treatment or removal of conduit.
Thromboembolic disease	Deep venous thrombosis or pulmonary embolus.
Infectious	
Pneumonia	New infiltrate on chest radiograph and positive tracheal aspirate cultures that requires antibiotic treatment.
Empyema	Pleural effusion on chest radiograph and positive culture of aspirated specimen that requires antibiotic and radiological or surgical treatment.
Abscess	Intra-thoracic (mediastinal) or intra-abdominal abscess confirmed by radiology with positive culture of aspirated specimen that requires antibiotic, radiological, or surgical treatment.
Wound infection	Erythematous wound, with effluent of pus and/or positive culture that requires opening of wound and antibiotics.
Gastrointestinal	Stool culture positive for microbial pathogens that requires antibiotic treatment.
Urinary tract	Positive urine culture and urine sediment that require antibiotic treatment.
Bacteremia	Blood samples showing positive growth and/or positive Gram stain, not reflecting colonization that requires antibiotic treatment.

Patients could suffer from multiple complications simultaneously

### Statistical Analysis

Patients were categorized into two groups, i.e., patients developing complications and without complications. In addition, to translate the results to clinical recommendations and to reflect complication severity, patients with post-operative complications were categorized into three mutually exclusive complication groups. Patients could either have purely surgical complications, purely infectious complications, or combined surgical and infectious complications. We studied biomarker levels at days 0–3 and their fractional change (∆) at day 3, i.e., day 3 divided by day 0 biomarker levels. Since most symptoms of complications appear after post-operative day 3, the levels measured between days 0 and 3 were considered early diagnostic for complications presenting between days 4 and 10. We used IBM SPSS statistics for Windows version 20 (IBM SPSS, Chicago, IL, USA) to analyze the data, except for analyzing the area under the receiver operating characteristics curve (AUROC). We present data as median (inter-quartile range) since many continuous data were non-normally distributed (Kolmogorov-Smirnov test, *P* < 0.05). We used a Kruskal-Wallis test and Mann-Whitney *U* test to study group differences in continuous variables and the *X* ^2^ or Fisher exact test for categorical variables. To evaluate the early diagnostic value of biomarker levels for groups, we calculated the AUROCs, for which non-Gaussian data were logarithmically transformed. Only the AUROC analyses were performed using MedCalc for Windows, version 13 (MedCalc Software, Ostend, Belgium). We considered an AUROC ≥ 0.70 as clinically relevant. The optimal diagnostic cutoff value was calculated as suggested by Zweig and Campbell.^25^ To calculate the optimal criterion, this method takes the disease prevalence and cost of true- and false-positive and true- and false-negative decisions into account.^25^ The Holm-Bonferroni method was used to correct for multiple testing.^26^ We used multiple logistic regression with backward selection of logarithmically transformed biomarker levels to study their interdependency for the diagnosis of post-operative complications in general, complication subtypes, and anastomotic leakage. We performed the Hosmer-Lemeshow test to evaluate the goodness of fit. All tests were two-sided, and *P* values < 0.05 were considered statistically significant; exact *P* values are given unless < 0.001.

## Results

Twenty-eight patients (62 %) suffered from a post-operative complication, of whom 5 had a surgical complication, 14 an infectious complication, and 9 combined surgical/infectious complications (Table 2). The manifestation of post-operative complications was on day 3 or later in all patients, and in 92 % of cases, complications presented on day 4 or later. Patients developing combined surgical/infectious complications had more complications than patients in the other complication groups (3 vs. 1 complication). Table 3 shows baseline characteristics for patients developing complications and without complications. The number of female patients was higher in the infectious and combined surgical/infectious than that in the surgical complication group. Almost all patients suffered from SIRS at some point during the first 10 days post-operatively. Patients developing infectious complications had received antibiotics less often than the other patient groups. Patients suffering surgical or combined complications had a longer hospital stay than patients with an uncomplicated recovery (*P* = 0.03 and *P* = 0.02, respectively). All patients survived until 30 days post-operatively. The preoperative World Health Organization (WHO) performance score and pulmonary function tests were not predictive of post-operative complications. To avoid major overlap, we do not separately report the baseline characteristics of patients with versus without anastomotic leak.

**Table 2:** Complications up to 10 days post-esophagectomy

Surgical complication (*N* = 5)	Infectious complication (*N* = 14)	Combined surgical/infectious complication (*N* = 9)
Anastomotic leak	Pneumonia, wound infection	Anastomotic leak, chyle leak, wound infection
Anastomotic leak	Pneumonia, wound infection	Anastomotic leak, pneumonia, wound infection
Pleural effusion, chyle leak	Pneumonia	Anastomotic leak, pneumonia
Chyle leak	Pneumonia	Anastomotic leak, abscess, wound infection
Chyle leak	Pneumonia	Anastomotic leak, pleural effusion, abscess, wound infection
	Pneumonia	Anastomotic leak, pneumonia
	Pneumonia	Anastomotic leak, wound infection, pneumonia, empyema
	Pneumonia	Anastomotic leak, pneumonia, empyema
	Pneumonia	Chyle leak, abscess, pneumonia
	Wound infection	
	Wound infection	
	Wound infection	
	Urinary tract infection	
	Urinary tract infection	

Complications presented no sooner than day 3; in 92 % of cases, complications presented on day 4 or after

**Table 3:** Baseline characteristics

	Uncomplicated			Complicated			
	(*N* = 17)	(*N* = 28)	P^1^	Surgical (*N* = 5)	Infectious (*N* = 14)	Combined surgical/infectious (*N* = 9)	P^2^
Sex (M)	16 (94)	23 (82)	0.39	2 (40)	12 (86)	9 (100)	0.009
Age (years)	62 (14)	63 (17)	0.52	60 (15)	65 (14)	63 (20)	0.89
BMI (cm^2^/kg)	27.8 (4.7)	23.8 (5.0)	0.05	22.7 (8.3)	25.3 (6.1)	23.5 (2.9)	0.12
WHO performance score							
0	7 (41)	14 (50)	0.57	7 (50)	2 (40)	5 (56)	0.57
1	10 (59)	14 (50)		7 (50)	3 (60)	4 (44)	
Preoperative pulmonary function							
FEV1 (% predicted)	113 (27)	98 (21)	0.04	96 (27)	100 (1)	94 (22)	0.19
VC (% predicted)	112 (28)	111 (16)	1.00	115 (17)	108 (2)	106 (22)	0.58
ASA class							
I	2 (12)	10 (11)	0.86	2 (40)	0	1 (11)	0.08
II	13 (77)	20 (71)		1 (20)	11 (79)	8 (89)	
III	2 (12)	5 (18)		2 (40)	3 (21)	0	
P-POSSUM score	35 (10)	34 (5)	0.50	34 (3)	33 (8)	35 (3)	0.70
Cell type							
Squamous cell carcinoma	2 (12)	9 (32)	0.28	4 (29)	2 (40)	3 (33)	0.85
Adenocarcinoma	13 (77)	18 (64)		6 (64)	3 (60)	6 (67)	
Small cell neuroendocrine carcinoma	1 (6)	1 (4)		1 (6)	0	0	
Miscellaneous	1 (6)	0		0	0	0	
Clinical stage							
T							
1	1 (6)	0	0.10	0	0	0	0.26
2	2 (12)	5 (18)		2 (14)	1 (20)	2 (22)	
3	11 (65)	19 (68)		12 (86)	3 (60)	4 (44)	
4	3 (20)	0		0	0	0	
Unknown	0	4 (14)		0	1 (20)	3 (33)	
N							
0	5 (29)	9 (32)	0.67	5 (36)	2 (40)	2 (22)	0.53
1	5 (29)	9 (32)		3 (21)	2 (40)	4 (44)	
2	6 (35)	9 (32)		6 (43)	1 (20)	2 (22)	
3	1 (6)	0		0	0	0	
Unknown	0	1 (4)		0	0	1(11)	
M							
0	17 (100)	26 (93)	0.52	14 (100)	5 (100)	7 (78)	na
Unknown	0	2 (7)		0	0	2 (22)	
Neoadjuvant chemoradiotherapy	14 (82)	26 (93)	0.35	5 (100)	13 (93)	8 (89)	0.66
Surgical approach							
TH	6 (35)	10 (36)	1.00	2 (40)	5 (36)	3 (33)	1.00
TT	11 (65)	18 (64)		3 (60)	9 (64)	6 (67)	
Open procedure	16 (94)	25 (59)	1.00	12 (86)	5 (100)	8 (89)	0.74
Laparoscopic procedure	1 (6)	3 (11)		2 (14)	0	1 (11)	
Hand sewn end-to-end	7 (41)	17 (61)	0.23	8 (57)	3 (60)	6 (67)	0.61
Semimechanical side-to-end	10 (59)	11 (39)		6 (43)	2 (40)	3 (33)	
Operation duration (min)	414 (186)	383 (136)	0.40	383 (171)	382 (140)	410 (156)	0.79
Blood loss (mL)	1000 (800)	675 (869)	0.33	600 (765)	725 (794)	700 (960)	0.33
APACHE II score	8 (5)	8 (3)	0.47	8 (2)	7 (6)	9 (4)	0.82
SOFA score							
Day 0	7 (2)	6 (4)	0.36	4 (3)	5 (3)	5(2)	1.00
Day 1	5 (3)	4 (5)	0.18	5 (2)	4 (6)	4 (2)	0.47
Day 2	3 (1)	3 (4)	0.92	3 (4)	2 (5)	4 (2)	0.97
Day 3	1 (2)	1 (4)	0.15	3 (2)	2 (3)	3 (5)	0.26
SIRS (days 0–10)	13 (77)	27 (96)	0.06	5 (100)	14 (100)	8 (89)	0.17
Sepsis (days 0–10)	0	17 (64)	< 0.001	0	11 (79)	6 (67)	< 0.001
Septic shock (days 0–10)	0	6 (21)	0.07	0	3 (21)	3 (33)	0.06
Prophylactic antibiotics i.o.	17 (100)	22 (100)	na	5 (100)	14 (100)	9 (100)	na
Antibiotics received (days 0–10)	4 (24)	20 (71)	0.002	10 (71)	3 (66)	7 (78)	0.02
Microbiology							
*Enterobacteriaceae*	0	7 (25)	0.03	0	5 (36)	2 (22)	0.03
*Pseudomonaceae*	0	5 (18)	0.14	0	3 (21)	2 (22)	0.15
*Staphylococcaceae*	0	1 (4)	1.00	0	1 (7)	0	0.50
*Streptococcaceae*	0	1 (4)	1.00	0	0	1 (11)	0.25
Miscellaneous	0	6 (21)	0.07	0	3 (21)	3 (33)	0.06
Vasopressor need (days 0–10)	7 (40)	12 (43)	0.91	2 (40)	5 (36)	5 (56)	0.82
ICU days	3 (1)	3 (1)	0.64	3 (2)	4 (2)	3 (5)	0.43
In hospital days	12 (6)	16 (10)	0.007	20 (12)	15 (6)	19 (12)	0.02
30-day mortality	0	0	na	0	0	0	na

Median (inter-quartile range), number (percentage), where appropriate; P^1^ comparison of uncomplicated vs. complicated patients by Mann-Whitney *U* or Fisher’s exact test where appropriate. P^2^ comparison of uncomplicated patient and all three complication groups by Kruskal-Wallis *H* or *X* ^2^ test, where appropriate

*APACHE* Acute Physiology and Chronic Health Evaluation, *ASA class* American Society of Anesthesiology physical status classification, *BMI* body mass index, *FEV1* forced expiratory volume in 1 s, *VC* vital capacity, *ICU* intensive care unit, *i.o.* intra-operatively, *m* male, *na* not applicable, *P-POSSUM* Portsmouth Physiological and Operative Severity Score for the enUmeration of Mortality and morbidity, *SIRS* Systemic Inflammatory Response Syndrome, *SOFA* Sequential Organ Failure Assessment, *TH* transhiatal, *TT* transthoracic, *WHO* World Health Organization

### Biomarker Levels Prior to Diagnosis of Complications

**Fig. 1:**
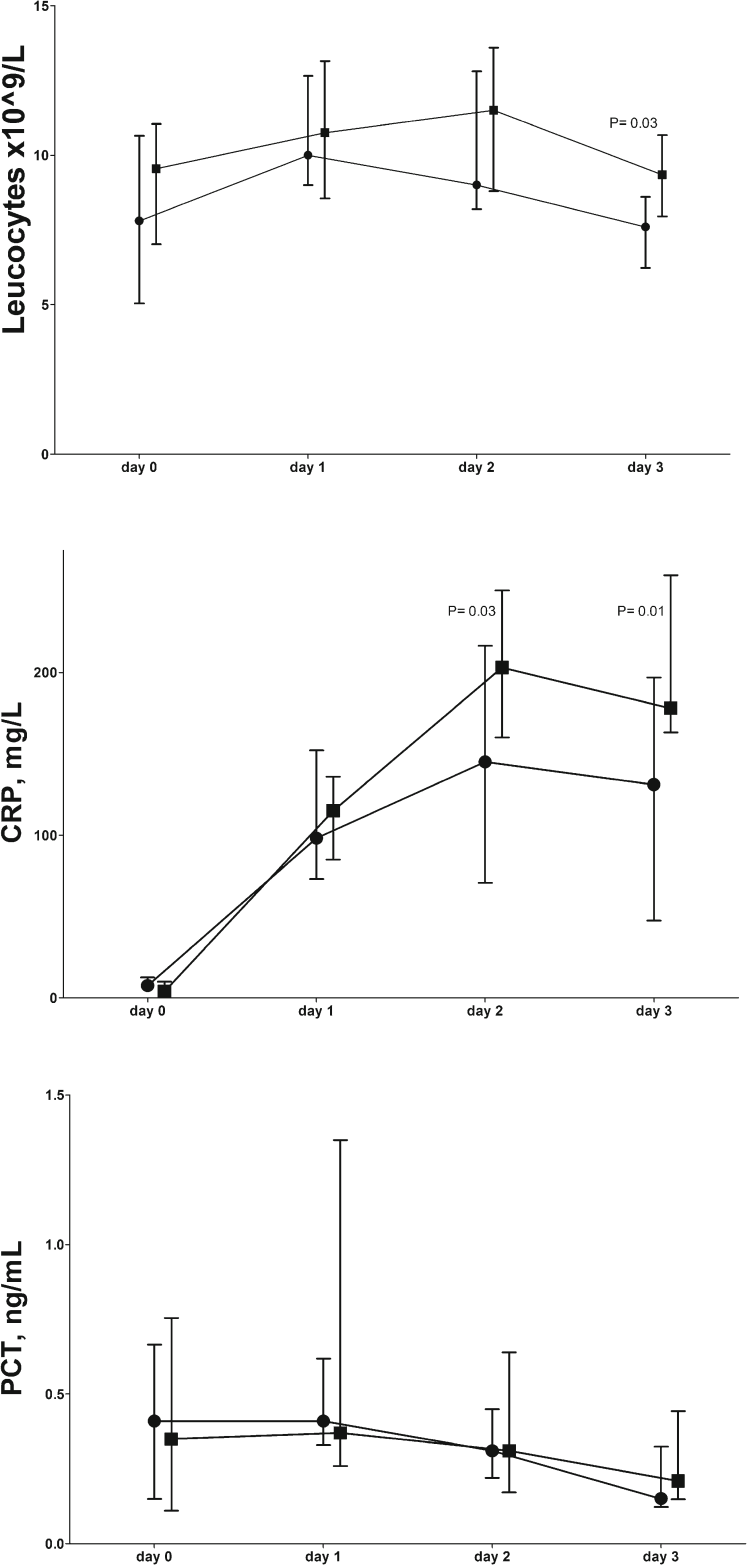
Early leukocyte and plasma biomarker levels (median and inter-quartile range) for complications up to 10 days after elective esophagectomy. Without complications (*N* = 17) (*circle*), with complications (*N* = 28) (*square*). *CRP* C-reactive protein, *PCT* procalcitonin. *P* values refer to Mann-Whitney *U* test

Figure 1 shows the data (days 0–3) for patients developing any complication and those without complications. Only statistically significant AUROC values are presented in Table 4. The day 3 leukocyte counts were higher in patients developing any complication than in those without, but the optimal cutoff value in AUROC was below the upper limit of the normal range. The day 2 and 3 CRP levels and their rise were higher in patients developing complications than in those without and had diagnostic value with high sensitivities of optimal cutoff values. The fractional change of CRP levels on day 3 vs. day 0 in patients developing complications was 46 (91) and in patients without complications 19 (27), *P* = 0.04. PCT levels could not discriminate between patients developing any type of complication and those without.

**Table 4:** Diagnostic values of biomarkers (days 0–3) for complications (up to day 10)

	Cutoff	AUROC	*P* value	SN	SP	PPV	NPV
Diagnostic values for any complication							
Leukocytes day 3	7.9 × 10^9^/L	0.71	0.02	75	64	78	60
CRP day 2	100 mg/L	0.71	0.04	100	36	74	100
CRP day 3	68 mg/L	0.75	0.006	100	43	75	100
∆ CRP days 0–3	23	0.75	0.01	75	78	86	64
Diagnostic values for combined surgical/infectious complications							
CRP day 3	316 mg/L	0.80	< 0.001	0	100	-	84
∆ CRP days 0–3	81	0.77	0.008	40	90	50	86
PCT day 3	1.15 ng/mL	0.86	< 0.001	38	100	100	81
Diagnostic values for anastomotic leak							
CRP day 3	229 mg/L	0.78	0.002	71	84	50	93
∆ CRP days 0–3	55	0.82	< 0.001	80	80	50	94
PCT day 1	1.82 ng/mL	0.76	0.005	22	100	100	83
PCT day 3	0.35 ng/mL	0.86	< 0.001	67	80	55	87

*AUROC* area under the receiver operating characteristics curve, *CRP* C-reactive protein, *NPV* negative predictive value, *PCT* procalcitonin, *PPV* positive predictive value, *SN* sensitivity, *SP* specificity, *∆* fractional change (day 3 divided by day 0 value)

### Biomarker Levels Prior to Diagnosis of Complication Subtypes

**Fig. 2:**
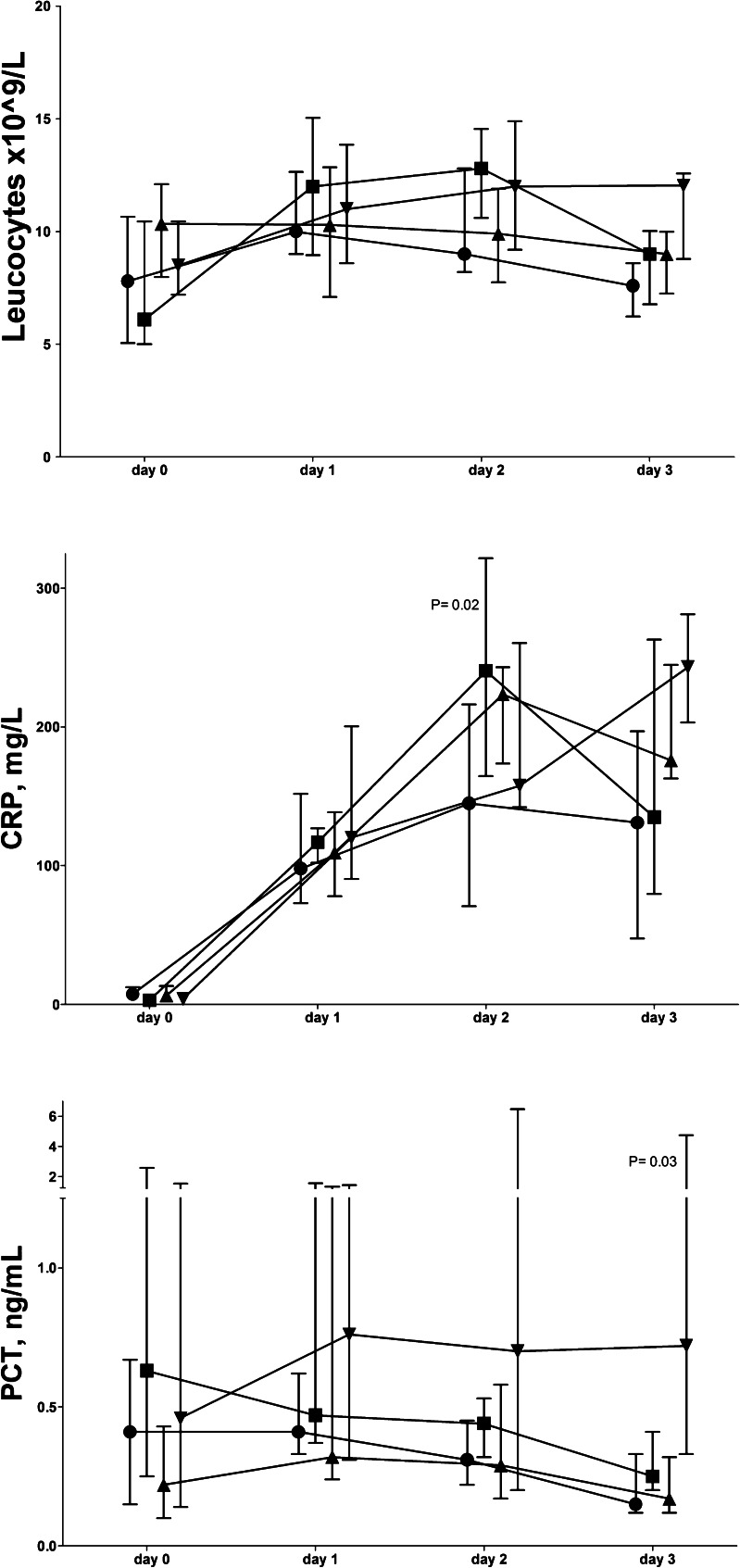
Early leukocyte and plasma biomarker levels (median and inter-quartile range) for complications up to 10 days after elective esophagectomy. Without complications (*N* = 17) (*circle*), surgical complications (*N* = 5) (*square*), infectious complications (*N* = 14) (*triangle*), combined surgical/infectious complications (*N* = 9) (*inverted triangle*). *CRP* C-reactive protein, *PCT* procalcitonin. *P* values refer to Kruskal-Wallis test

Figure 2 shows the data (days 0–3) for complication subtypes. On day 3, leukocyte counts were higher in patients with combined surgical/infectious complications than those without complications (*P* = 0.01). The CRP levels on days 2 and 3 were higher in patients with infectious complications than in those without complications (*P* = 0.03), whereas day 3 CRP levels were higher in patients with combined complications than in those without (*P* = 0.01). The fractional increase in CRP was higher in patients developing combined complications, by 76 (41), than in patients without complications, by 19 (27), *P* = 0.02. On day 3, PCT levels were higher in patients developing combined surgical/infectious complications than in those without complications and developing surgical or infectious complications (*P* = 0.009).

**Fig. 3:**
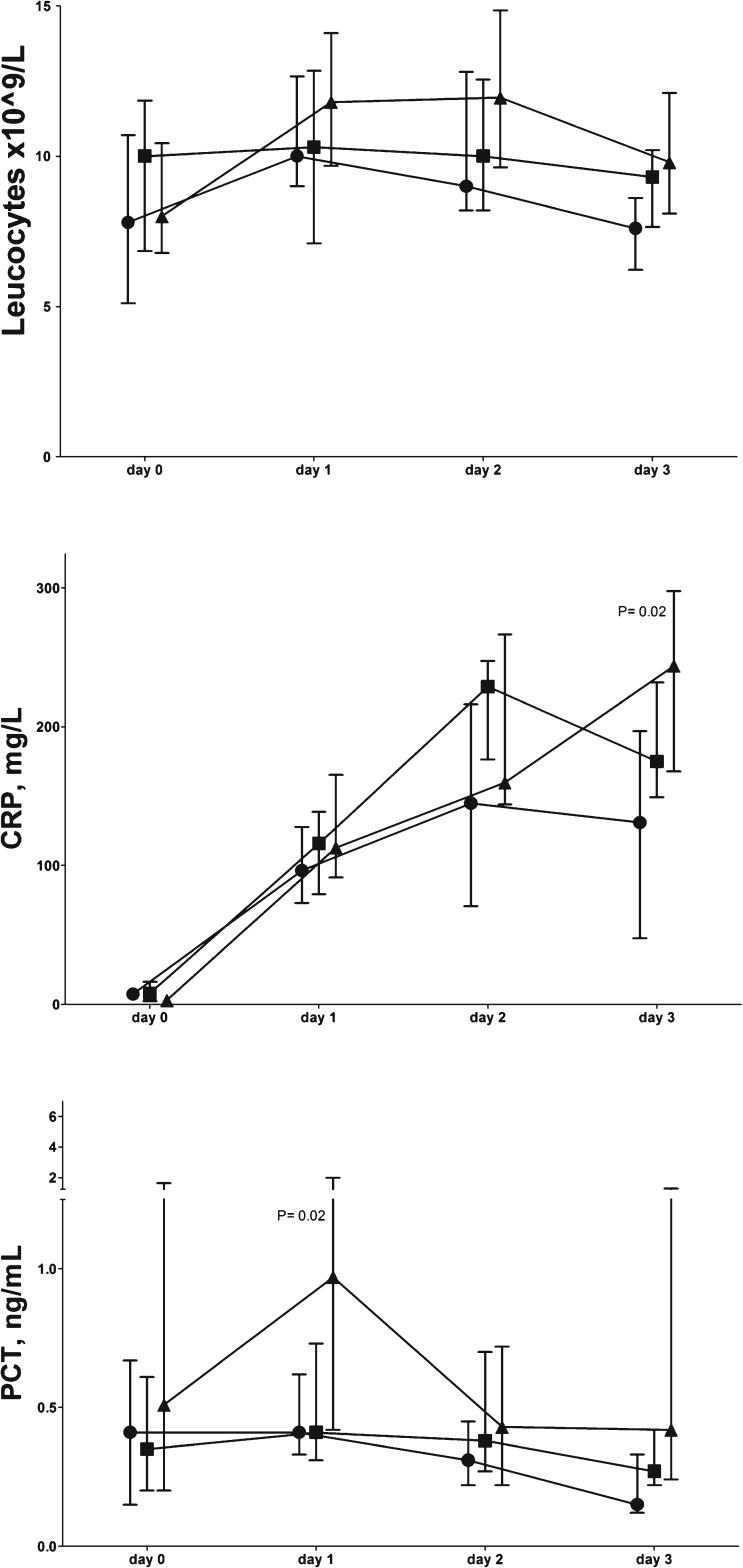
Early leukocyte and plasma biomarker levels (median and inter-quartile range) for complications up to 10 days after elective esophagectomy. Without complications (*N* = 17) (*circle*), with other complications (*N* = 18) (*square*), with anastomotic leakage (*N* = 10) (*triangle*), *CRP* C-reactive protein, *PCT* procalcitonin. *P* values refer to Kruskal-Wallis test

Figure 3 shows the data (days 0–3) for patients with anastomotic leakage versus patients with other complications or without complications. On day 2, CRP levels were higher in patients developing other complications than anastomotic leakage compared to those without complications (*P* = 0.02). However, on day 3, the CRP levels were higher in patients developing anastomotic leak compared to all patients (*N* = 35) without leakage (*P* = 0.02). The PCT levels on days 1 and 3 were higher in patients with anastomotic leakage compared to all patients (*N* = 35) without leakage (*P* = 0.02 and *P* = 0.03, respectively). Furthermore, the day 1 PCT levels were higher in patients with anastomotic leakage vs. other complications (*P* = 0.02).

The diagnostic value of elevated day 3 PCT preceded the clinical diagnosis of combined complications and anastomotic leakage, as did the day 3 CRP levels and their fractional changes (Table 4). The diagnostic value of high day 1 PCT levels already preceded anastomotic leakage, however.

### Multiple Logistic Regression

On day 2, CRP was diagnostic for developing complications independently from leukocytes and PCT (*P* = 0.038, Hosmer-Lemeshow *X* ^2^ 5.22, *df* 8, *P* = 0.734). On day 3, CRP levels were diagnostic for developing anastomotic leakage independently from leukocytes and PCT (*P* = 0.032, Hosmer-Lemeshow *X* ^2^ 8.8, *df* 7, *P* = 0.268). On day 1, PCT was diagnostic for developing anastomotic leak independently from leukocytes and CRP (*P* = 0.016, Hosmer-Lemeshow *X* ^2^ 8.064, *df* 8, *P* = 0.427).

## Discussion

This relatively small study suggests that elevated CRP levels are a sensitive marker of complications developing post-esophagectomy, whereas elevated PCT levels may specifically indicate the development of more severe combined surgical/infectious complications, mainly associated with anastomotic leakage, within 3 to 10 days post-esophagectomy.

Even though all patients had low ASA classification, P-POSSUM, and APACHE II scores, 62 % had early post-operative complications. There were no fatalities within 30 days post-operatively. The preoperative risk assessment scores were comparable between groups and thus unsuitable for indicating development of a complicated post-operative clinical course. Although the complication rate appears relatively high, the rate and type are in line with the literature.^2^–^9^,^11^–^13^,^22^,^27^,^28^ Up till now, there are no uniformly accepted guidelines for reporting of post-operative complications, and a recent systematic review has shown a wide range in definitions hampering interpretation of study results.^4^ The difficulty in uniform, mutually exclusive complication categories makes interpretation and comparison of studies difficult. We grouped complications since they represent different conditions and associated severities, whereas the group was too small to attempt to discriminate between individual complications. Patients who developed combined surgical/infectious complications had more complications simultaneously than patients in the other complication groups. Furthermore, their hospital stay was longer than of patients with infectious complications or without complications.

This is the first study trying to discriminate among early post-operative complication types by using CRP and PCT. All complications presented on day 3 or later, and in 92 % of cases, complications presented on day 4 or later. We may argue that since the cutoff values of day 2 and 3 biomarker levels precede the clinical symptoms and diagnosis of complication, they are predictive in time. The elevation of CRP levels in patients without complications is also comparable to that reported before.^6^,^8^,^9^,^11^–^13^ Studies reported high PCT levels, as in our study, after esophagectomy or other extensive gastrointestinal surgeries irrespective of complications,^1^,^15^,^19^ and high PCT levels, albeit not more elevated than CRP, in major anastomotic leakage after colorectal surgery.^16^,^18^ Based on our observations and those of others,^6^,^7^,^9^,^12^,^15^,^17^ one may thus hypothesize that both CRP and PCT increase following a surgical host response, but that PCT follows a more severe manifestation of this response, particularly when associated with surgical/infectious complications. Indeed, we could not discriminate infectious complications from surgical complications by use of PCT or CRP, but PCT rather than CRP was able to identify patients at risk for more severe combined complications after esophagectomy.

In detail, CRP levels on days 2 and 3 were diagnostic for any complication presenting between days 3 and 10, independent of preoperative risk assessment score and SIRS criteria. The calculated sensitivity and specificity are similar to those reported in some previous studies,^9^,^10^ but in slight contrast to others who found a diagnostic value of CRP no sooner than on post-operative day 4^8^,^11^,^12^ or no diagnostic value at all for anastomotic leakage or infectious complications.^6^,^13^ In our study, neither CRP levels nor fractional increases could differentiate between complication groups, limiting the use of CRP levels for early recognition of complication subtypes. The low specificity and modest positive predictive value calculated from the AUROC suggest that the use of an elevated CRP alone as an indicator of developing complications post-esophagectomy may lead to antibiotic overtreatment, amongst others, if considered specific for infection.

Plasma PCT levels have been studied and compared with CRP in patients after major surgery and trauma, but the results are inconclusive.^14^–^18^,^20^ So far, one study on post-esophagectomy showed a diagnostic value of PCT for development of sepsis^11^ and another one for infectious complications.^13^ We found an early diagnostic value of day 3 PCT levels for combined surgical/infectious complications presenting between days 3 and 10 independently from preoperative risk assessment scores, but not of infectious complications alone. PCT was the only marker of help in the early diagnosis of more severe complications and the earliest one to recognize anastomotic leakage, the most common combined surgical/infectious complication. Even though the AUROC of day 3 CRP was statistically significant for combined complications, the marker level had little positive predictive value. The positive predictive value of PCT levels is higher, and PCT is therefore preferred over CRP for diagnosis of combined complications. As a result, elevated PCT levels at the cutoff levels presented could guide additional diagnostics and start of empirical antibiotics before full-blown presentation of complications post-esophagectomy.

The leukocyte counts peaked around the upper limit of normal on day 2 in agreement with some studies.^6^,^8^,^10^,^11^ This relatively low leukocyte peak count could be explained by neoadjuvant chemotherapy in the majority of patients. Some investigators found a moderately elevated leukocyte count on days 2 to 5 to predict anastomotic leak and infectious complications.^8^,^10^,^11^ The leukocyte count in our study did not discriminate between surgical, infectious, or combined complications and is therefore not useful for this purpose, as in other studies.^6^,^13^ We included this SIRS criterion for reasons of comparison with CRP and PCT.

The limitations of this proof of principle study include its relatively small and heterogeneous sample size. Furthermore, little is known about the effects of neoadjuvant chemoradiotherapy on biomarker release and kinetics. However, almost all patients in our study received such treatment and predictive values of biomarkers were maintained. There is no difference in effect on post-operative CRP and PCT values reported between laparoscopic and open surgery or between the transhiatal and transthoracic approaches, respectively.^1^,^29^

### Conclusion

An increasing or high CRP level within 3 days after elective esophagectomy may contribute to the early diagnosis of any post-operative complications presenting between post-operative days 3 and 10, independent of the preoperative risk assessment scores. Elevated PCT levels may specifically indicate severe combined surgical/infectious complications, mainly associated with anastomotic leakage, but may not recognize infectious complications alone. Nevertheless, PCT rather than CRP might be used for decisions on additional diagnostics and empirical antibiotic treatment in these patients.

## Abbreviations

AUROC: Area under the receiver operator characteristics curve
APACHE II score: Acute Physiology and Chronic Health Evaluation II score
ASA classification: American Society of Anesthesiologists classification
CRP: C-reactive protein
CV: Coefficient of variation
ICU: Intensive care unit
PCT: Procalcitonin
P-POSSUM: Portsmouth Physiological and Operative Severity Score for the enUmeration of Mortality and morbidity
SIRS: Systemic inflammatory response syndrome
SOFA score: Sequential Organ Failure Assessment score
WHO: World Health Organization
